# Safety and Efficacy of Poly‐L‐Lactic Acid Filler (HYZ vs. Löviselle) Injection for Nasolabial Fold Correction: A Double‐Blind, Non‐Inferiority, Randomized, Controlled Trial

**DOI:** 10.1111/jocd.71260

**Published:** 2026-10-25

**Authors:** Zhanhong Li, Jingfang Sun, Lianbo Zhang, Aili Cui, Sha Huang, Yaowu Dong, Haibo Guo, Yongmei Shi, Xiaoyi Qiu, Qiuting Huang, Xiang Gao, Yuan Yuan, Yanqi Ye, Xiao Wang, Liang Chen, Chongguo Liang, Liwei Wu, Junlong Guo, Xiaojuan Zhang, Wenting Chen, Ping Yu, Jing Lv, Fengsheng Che, Qiong Meng

**Affiliations:** ^1^ Sihuan Pharmaceutical Holdings Group Ltd. Beijing China; ^2^ Meiyan Space, Sihuan Pharmaceutical Holdings Group Ltd. Beijing China; ^3^ Department of Plastic Surgery China‐Japan Union Hospital of Jilin University Jilin China; ^4^ Department of Medical Cosmetology Yanbian University Hospital Jilin China; ^5^ Department of Plastic Surgery, Shulan (Hangzhou) Hospital, Shulan International Medical College Zhejiang Shuren University Hangzhou China; ^6^ Department of Otolaryngology Tonghua Central Hospital Tonghua China; ^7^ Beijing Aohe Pharmaceutical Research Institute Co., Ltd. Beijing China; ^8^ Department of Cosmetic Dermatology Beijing MYLIKE Medical Cosmetic Hospital Beijing China; ^9^ Department of Cosmetic Dermatology Shenzhen Xi'en Medical Cosmetic Clinic Shenzhen China; ^10^ Department of Cosmetic Dermatology Anhui Haobai Medical Cosmetic Clinic Hefei China; ^11^ Department of Cosmetic Dermatology Hainan Yishangmei Medical Cosmetic Clinic Haikou China; ^12^ Department of Cosmetic Dermatology Wenzhou Peace International Hospital Wenzhou China; ^13^ Department of Cosmetic Dermatology Qingdao Doctor Medical Cosmetic Hospital Qingdao China; ^14^ Department of Cosmetic Dermatology Qingdao LORD Medical Cosmetic Hospital Qingdao China; ^15^ Department of Cosmetic Dermatology Shenzhen MYLIKE Medical Cosmetic Hospital Shenzhen China; ^16^ Department of Cosmetic Dermatology Sichuan Huamei Zixin Plastic Surgery Hospital Chengdu China; ^17^ Department of Cosmetic Dermatology Chongqing Xinfu Medical Cosmetic Clinic Chongqing China; ^18^ Hunan University of Medicine Huaihua China; ^19^ School of Pharmaceutical Sciences Sun Yat‐Sen University Guangzhou China

**Keywords:** nasolabial fold, non‐inferiority trial, poly‐L‐lactic acid

## Abstract

**Background:**

Poly‐L‐lactic acid (PLLA) is an established soft‐tissue filler for correcting nasolabial folds (NLFs), offering durable results and high patient satisfaction. This study evaluated the efficacy and safety of a novel PLLA filler (HYZ) for this indication.

**Methods:**

In this prospective, multicenter, randomized, double‐blind, non‐inferiority clinical trial, 176 subjects were randomly assigned to two groups, were randomized to receive three sessions of either HYZ (experimental group) or Löviselle (control group) injections at 3‐week intervals. Efficacy was assessed using the Global Aesthetic Improvement Scale (GAIS) and the Wrinkle Severity Rating Score (WSRS). Safety was evaluated based on adverse events (AEs).

**Results:**

From baseline to Week 54, the HYZ group consistently showed higher WSRS response rates than the control group. At Week 54, the response rate was significantly higher in the HYZ group (54.02% vs. 36.36%, *p* < 0.05), meeting the predefined non‐inferiority margin (Δ = −10%). No significant between‐group differences were observed in GAIS response rates. The incidence of adverse events was comparable between the two groups.

**Conclusion:**

The novel PLLA filler (HYZ) was non‐inferior to the established product Löviselle in both efficacy and safety for NLF correction. These findings provide new clinical evidence supporting the continued development and optimization of PLLA‐based fillers.

## 1 Introduction

Nasolabial fold (NLF), extending from the lateral aspect of the alar base to the corner of the mouth, represents one of the most prominent aesthetic concerns in the mid‐to‐lower face [1]. Progressive deepening of the NLF is closely associated with age‐related synergistic changes, including descent of the malar fat pad, loss of subcutaneous soft‐tissue volume, and degradation of dermal elastic fibers and collagen architecture, reflecting a complex biological aging process [2, 3]. Pronounced NLFs often convey a fatigued, stern, or aged appearance, thereby generating substantial demand for aesthetic correction [4].

In recent years, a minimally invasive aesthetic paradigm characterized by reduced downtime, favorable safety profiles, and natural‐looking outcomes has increasingly dominated the global aesthetic medicine market [5]. Within this context, injectable soft‐tissue augmentation has emerged as a preferred option for patients seeking facial rejuvenation, owing to its procedural simplicity and limited invasiveness [6]. Among the available injectable materials, poly‐L‐lactic acid (PLLA), a biocompatible and biodegradable synthetic polymer, has attracted considerable clinical interest [7]. Unlike fillers that rely primarily on immediate volumization through physical space occupancy, PLLA exerts its effect by inducing a controlled foreign‐body response that stimulates endogenous collagen synthesis and remodeling over time [8, 9]. This gradual biological process typically unfolds over several months, resulting in progressive and physiologic restoration of soft‐tissue volume, with effects that are often more durable and natural in appearance than those achieved with conventional hyaluronic acid fillers [10].

The clinical efficacy and safety profile of PLLA fillers, such as Löviselle, in addressing midfacial volume deficit and improving NLF severity have been extensively validated through evidence‐based medicine [11]. Nevertheless, the clinical performance of PLLA formulations, including efficacy, safety, and ease of administration, is highly dependent on their microstructural properties, such as particle size and size distribution uniformity [12, 13]. Continuous material optimization is therefore essential to expand the product safety margins and improve user experience in clinical practice [14, 15].

HYZ is a novel PLLA filler designed with a narrowly controlled particle size range of 25–32 μm and an optimized particle size distribution to reduce aggregation tendency and improve reconstitution efficiency and injection stability. These material refinements are intended to deliver more consistent clinical outcomes and a smoother injection experience [16]. Based on these considerations, we conducted a prospective, large‐scale clinical study in a Chinese population to evaluate the efficacy and safety of HYZ for the correction of NLFs. Using Löviselle as the comparator, this study employed standardized efficacy endpoints and a structured follow‐up framework to directly compare the clinical performance of the two PLLA formulations under identical treatment conditions.

## 2 Methods

### 2.1 Subjects

The trial was conducted at four clinical centers in China. From February 10, 2022 to July 25, 2023, a total of 176 healthy participants aged 18 years or older were enrolled. Eligible participants presented with bilateral NLFs graded 2 or 3 on the Wrinkle Severity Rating Scale (WSRS). All participants provided written informed consent prior to study entry and agreed to comply with the study protocol. Participants acknowledged and accepted the requirement to refrain from undergoing any facial aesthetic procedures during the study period and committed to completing all scheduled treatments and follow‐up visits.

Exclusion criteria comprised: individuals who were pregnant, lactating, or planning pregnancy during the study period; a history of abnormal wound healing or scar formation; active facial skin conditions (e.g., infection, inflammation, ulceration, or malignant disease); known allergy to any component of the investigational product, gram‐positive bacterial proteins, or amide‐type local anesthetics; administration of facial fillers, surgical procedures, or laser/energy/chemical peel treatments in the past 6 months; use of immunosuppressants or systemic corticosteroids within 3 months prior to enrollment; ongoing anticoagulant therapy or a history of bleeding disorders, or use of anticoagulants or medications affecting coagulation within 14 days; severe systemic diseases including malignancy, autoimmune or connective tissue disorders, severe organ dysfunction, coagulopathy, or chronic neuromuscular disorders; and any other factors that could potentially affect the study, such as psychiatric disorders, substance abuse, or chronic infections.

Eligible participants were randomly assigned in a 1:1 ratio to either the control group (Löviselle injection) or the experimental group (HYZ injection). All participants received three injection sessions at 3‐week intervals. Follow‐up assessments were conducted at Weeks 12, 24, and 48 after the final injection.

### 2.2 Treatment

Prior to treatment, subjects received topical anesthesia with a compound lidocaine cream (Registration No. H20063466, Tongfang Pharmaceutical Group Co. Ltd., China) applied for 30–40 min. After removal of the anesthetic cream, the treatment area was disinfected with 5% iodophor and subsequently rinsed thoroughly with 0.9% normal saline to remove residual iodine. Subjects in the experimental group received HYZ injections (Registration No. 20253130831, Sihuan Pharmaceutical Holdings Group Ltd., Beijing, China), whereas those in the control group were treated with Löviselle (Registration No. 20213130276, Changchun Sinobiomaterials Co. Ltd., China). A total of three treatment sessions were administered at 3‐week intervals. Löviselle was reconstituted with 5 mL of sodium chloride injection and shaken for 1 min to facilitate dissolution, followed by a 30‐min standing period to allow air bubbles in the suspension to rise. In contrast, HYZ was reconstituted with 5 mL of sterile water for injection and required only 1 min of shaking before use to achieve a homogeneous suspension. For both products, the vial was gently shaken again immediately before injection to ensure uniform dispersion of the suspension. The investigational products were injected into the deep dermis or subcutaneous tissue using a 27G blunt needle. The total injected volume for bilateral NLFs did not exceed 3 mL per session. Immediately after injection, the treated areas were gently massaged, followed by intermittent application of cold compresses.

### 2.3 Efficacy Evaluation

Both the investigators and participants were blinded to treatment allocation throughout the study. The study products were prepared in advance, with all identifying label information concealed, and were subsequently provided to the investigators for administration. Neither the investigators nor the participants had access to any information or documentation that could potentially compromise the blinding status during the study. Standardized frontal and lateral facial photographs were obtained at baseline and at each follow‐up visit. Treatment efficacy was assessed using the Wrinkle Severity Rating Scale (WSRS) and the Global Aesthetic Improvement Scale (GAIS) [17, 18]. The primary endpoint was the WSRS response rate for bilateral NLFs at the follow‐up visit 48 weeks after the final injection (Week 54), defined as the proportion of participants in the treatment and control groups demonstrating improvement from baseline, as evaluated by an independent third‐party assessor. Secondary endpoints included the response rates of the GAIS and WSRS at Week 3, 6, 18, 30, and 54, based on investigator and participant assessments, as well as the WSRS response rates for bilateral NLFs at Week 18 (12 weeks after the final injection) and Week 30 (24 weeks after the final injection), as evaluated by independent third‐party assessors (Tables 1 and 2). A response was defined as an improvement of at least one grade on the WSRS or GAIS compared with baseline.

**TABLE 1:** Global Aesthetic Improvement Scale (GAIS).

Grade	Notes
3	Very much improved
2	Much improved
1	Improved
0	No change
−1	Worse

**TABLE 2:** Wrinkle Severity Rating Scale (WSRS).

Grade	Notes
0 (Note)	No visible wrinkles; normal, continuous skin texture
1 (Minimal)	Fine, shallow wrinkles with slight depressions
2 (Moderate)	Clearly visible wrinkles at rest that disappear upon skin stretching
3 (Severe)	Prominent, deep wrinkles; folds (< 2 mm) visible upon skin stretching
4 (Extreme)	Extremely deep wrinkles; visible V‐shaped folds (2–4 mm) persist upon stretching

### 2.4 Safety Evaluation

Baseline demographic data, including sex and age, were collected for participants in both groups. Participants underwent laboratory evaluations, including complete blood count, routine urinalysis, and assessments of hepatic and renal function, as well as monitoring of vital signs, including blood pressure, heart rate, and body temperature. Following each injection, participants were closely observed for local reactions at the injection sites, such as swelling, pain, and pruritus. All adverse events (AEs) and serious adverse events (SAEs) were documented and summarized according to the number of affected participants, incidence rates, and percentages.

### 2.5 Statistical Analysis

Three analysis populations were defined in this study: (1) the full analysis set (FAS) comprised all enrolled participants who received at least one treatment and underwent at least one post‐treatment efficacy assessment. Efficacy analyses were primarily based on the FAS. Missing data for the primary endpoint were imputed using the Worst Observation Carried Forward (WOCF) method. No imputation was applied to secondary efficacy endpoints. (2) The per‐protocol set (PPS) included participants who completed the study in compliance with the protocol and had evaluable data for the primary endpoint. The PPS analysis was conducted without missing data imputation and was used to assess the consistency and robustness of the efficacy results obtained from the FAS analysis. (3) The Safety Set (SS) consisted of all participants who received at least one administration of the investigational medical device. Statistical analysis was performed using SAS 9.4 software. All statistical tests were two‐sided, and *p* < 0.05 was considered statistically significant. Parameter estimates are presented with two‐sided 95% confidence intervals (CI). Quantitative variables were summarized using descriptive statistics, including the number of observations, mean, standard deviation, median, first quartile and third quartile. Categorical variables were described as numbers and percentages. Between‐group comparisons were performed using the *t*‐test or Wilcoxon rank‐sum test for quantitative variables, the Chi‐squared test or Fisher's exact test for categorical variables (if the Chi‐squared test is not applicable), and the Wilcoxon rank‐sum test or Cochran–Mantel–Haenszel (CMH) test for ordinal data.

## 3 Results

### 3.1 Subjects

A total of 176 subjects were enrolled in this trial and randomly assigned in a 1:1 ratio to the experimental group or the control group (Figure 1). At Week 48 after treatment, follow‐up was completed for 147 subjects, including 75 subjects in the experimental group and 72 subjects in the control group. During the study period, 29 subjects discontinued the trial prematurely, comprising 13 subjects in the experimental group and 16 subjects in the control group. All 176 enrolled subjects were included in the SS, with 88 subjects in each group. The FAS consisted of 175 subjects, including 87 subjects in the experimental group and 88 subjects in the control group. The PPS included 146 subjects, with 74 subjects in the experimental group and 72 subjects in the control group. The sample sizes for each set met the statistical requirements specified in the protocol.

**FIGURE 1:**
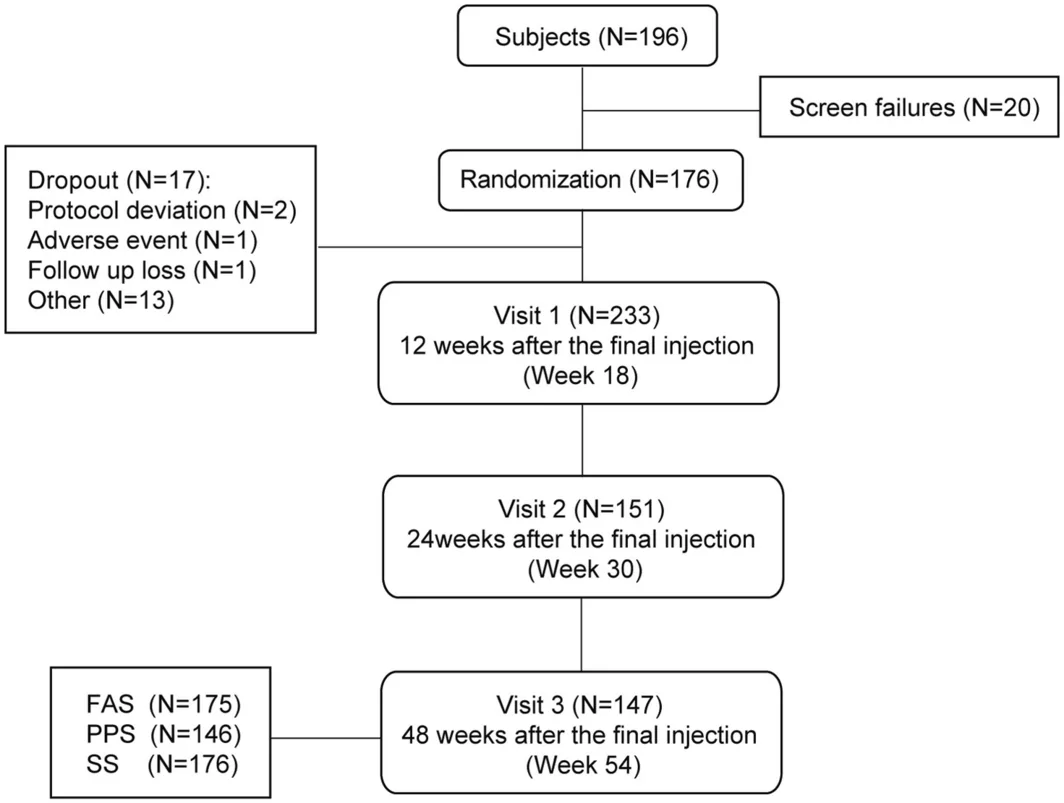
Flowchart of subjects distribution in the trial.

The demographic characteristics of subjects in the experimental group and the control group were summarized in Table 3, including age, sex, height, and body weight. In the experimental group, the mean age of subjects was 44.47 years, with a mean height of 162.53 cm and a mean body weight of 60.18 kg. Female subjects accounted for 81 cases (93.10%), while male participants accounted for 6 cases (6.90%). In the control group, the mean age was 44.74 years, the mean height was 161.26 cm, and the mean body weight was 57.61 kg; all participants in this group were female.

**TABLE 3:** Demographic data.

Parameter	Indicator	Experimental group	Control group	Statistic	*p*
Age (years)	*N* (Missing)	87 (0)	88 (0)	Wilcoxon rank‐sum test −0.2882	0.7732
	Mean	44.47	44.74		
	Median	42	44		
	Min, Max	25, 70	26, 66		
	IQR	15	17		
Gender	Female, *n* (%)	81 (93.10%)	88 (100.00%)	Fisher's exact test	0.0138
	Male, *n* (%)	6 (6.90%)	0 (0.00%)		
Height (cm)	*N* (Missing)	87 (0)	88 (0)	Wilcoxon rank‐sum test 1.2583	0.2083
	Mean	162.53	161.26		
	Median	162	160		
	Min, Max	150, 182	150, 175		
	IQR	8	7		
Weight (kg)	*N* (Missing)	87 (0)	88 (0)	Wilcoxon rank‐sum test 1.9771	0.0480
	Mean	60.18	57.61		
	Median	60	57.25		
	Min, Max	42, 90	42, 77.50		
	IQR	11	9		

### 3.2 Primary Efficacy Endpoint

Based on independent third‐party assessments, the experimental group demonstrated a significantly higher WSRS response rate than the control group at 48 weeks following the final injection (*p* < 0.05). The response rates for FAS and PPS were 54.02% and 63.51% in the experimental group, respectively, compared to 36.36% and 44.44% in the control group (Figure 2). The between‐group differences were 0.18 (95% CI: 0.0291, 0.3241) and 0.19 (95% CI: 0.0290, 0.3524), respectively. Since the lower limits of both confidence intervals were greater than the prespecified non‐inferiority margin (Δ = −10%), the efficacy of the experimental group was demonstrated to be non‐inferior to that of the control group. Figure 3 shows photographs of the subjects before and after injection with HYZ (at baseline, Week 3, 6, 18, 30, and 54).

**FIGURE 2:**
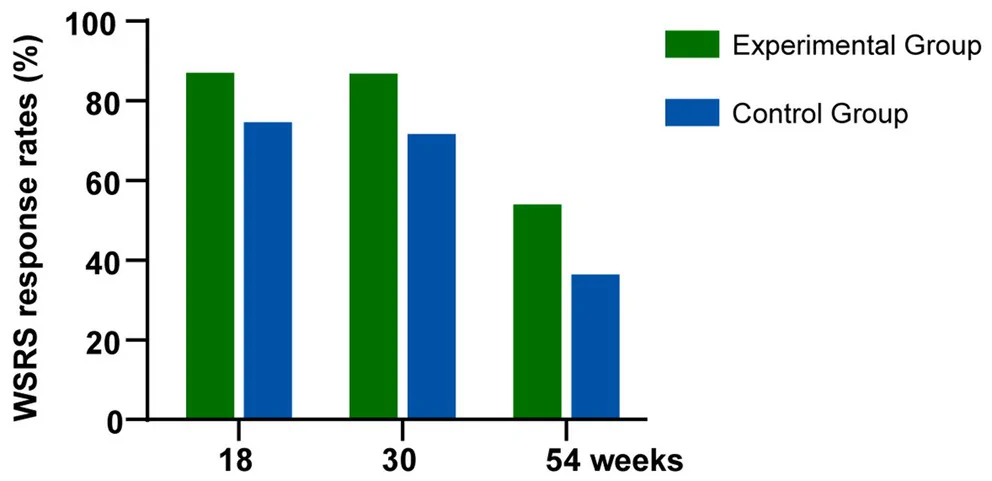
WSRS response rates at Week 18, 30, and 54 assessed by independent evaluators in the FAS population.

**FIGURE 3:**
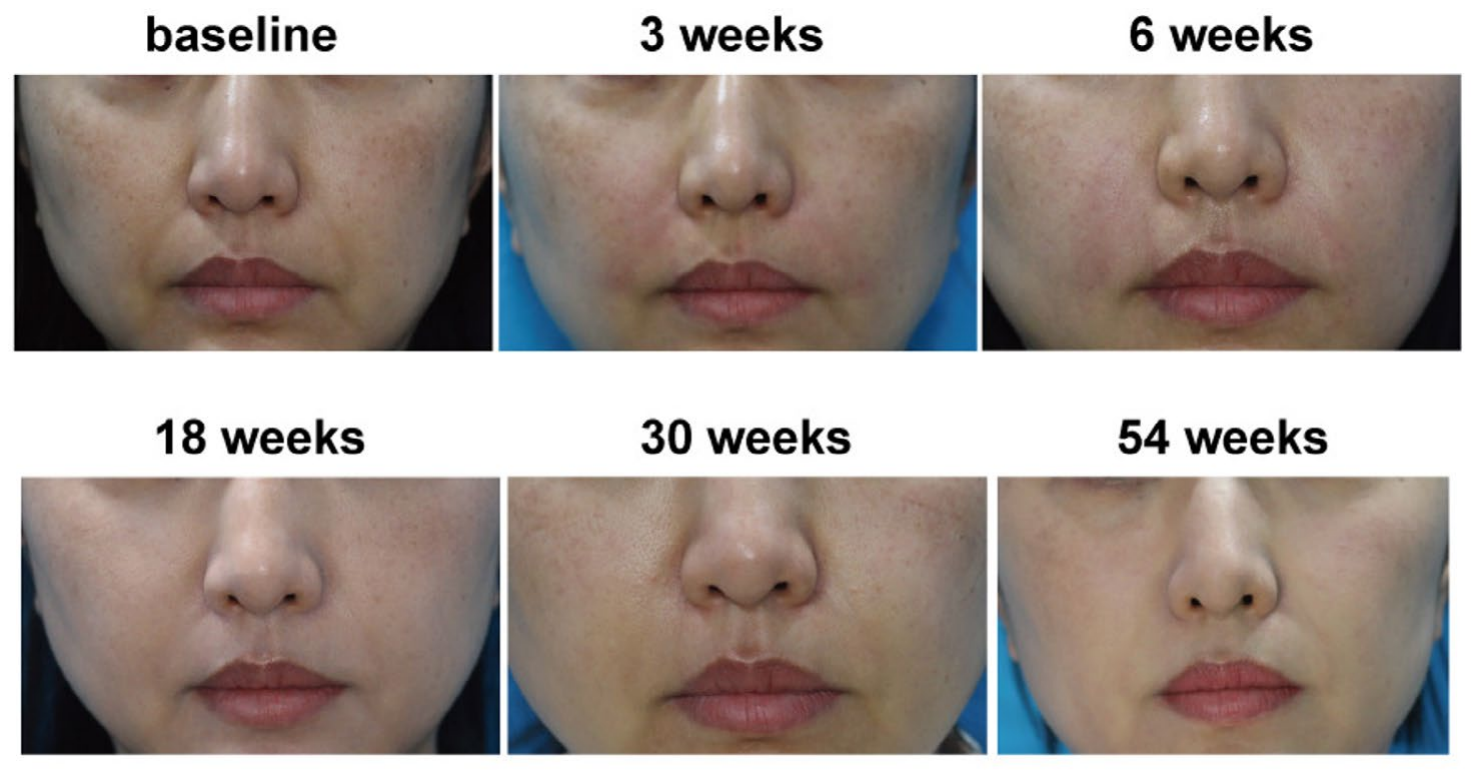
Clinical imaging records of representative cases receiving HYZ treatment. Images were taken at baseline, Week 3, 6, 18, 30, and 54 post‐treatment to show NLF improvements.

### 3.3 Secondary Efficacy Endpoint

Analyses of all secondary efficacy endpoints were performed based on the FAS population. According to assessments conducted by independent evaluators, the WSRS response rates at Week 18 were 87.01% in the experimental group and 74.67% in the control group, with no statistically significant difference between groups (*p* > 0.05). By Week 30, the corresponding response rates were 86.84% and 71.62%, respectively, with the experimental group demonstrating a significantly higher response rate than the control group (*p* < 0.05). Based on the investigator's assessment at Week 3, 6, 18, 30, and 54, the WSRS response rates in the experimental group were 92.68%, 92.41%, 80.52%, 80.26%, and 54.05%, respectively, whereas the corresponding rates in the control group were 86.42%, 87.34%, 81.33%, 75.68%, and 44.44% (Figure 4). Correspondingly, the GAIS response rates were 67.07%, 70.89%, 55.84%, 56.58%, and 39.19% in the experimental group, compared with 69.14%, 70.89%, 54.67%, 52.70%, and 25.00% in the control group. Subject's assessments were generally consistent with investigator assessments, both indicating that although response rates gradually declined over time following completion of the treatment course, a sustained long‐term therapeutic benefit was maintained. No statistically significant differences between groups were observed at any visit (*p* > 0.05).

**FIGURE 4:**
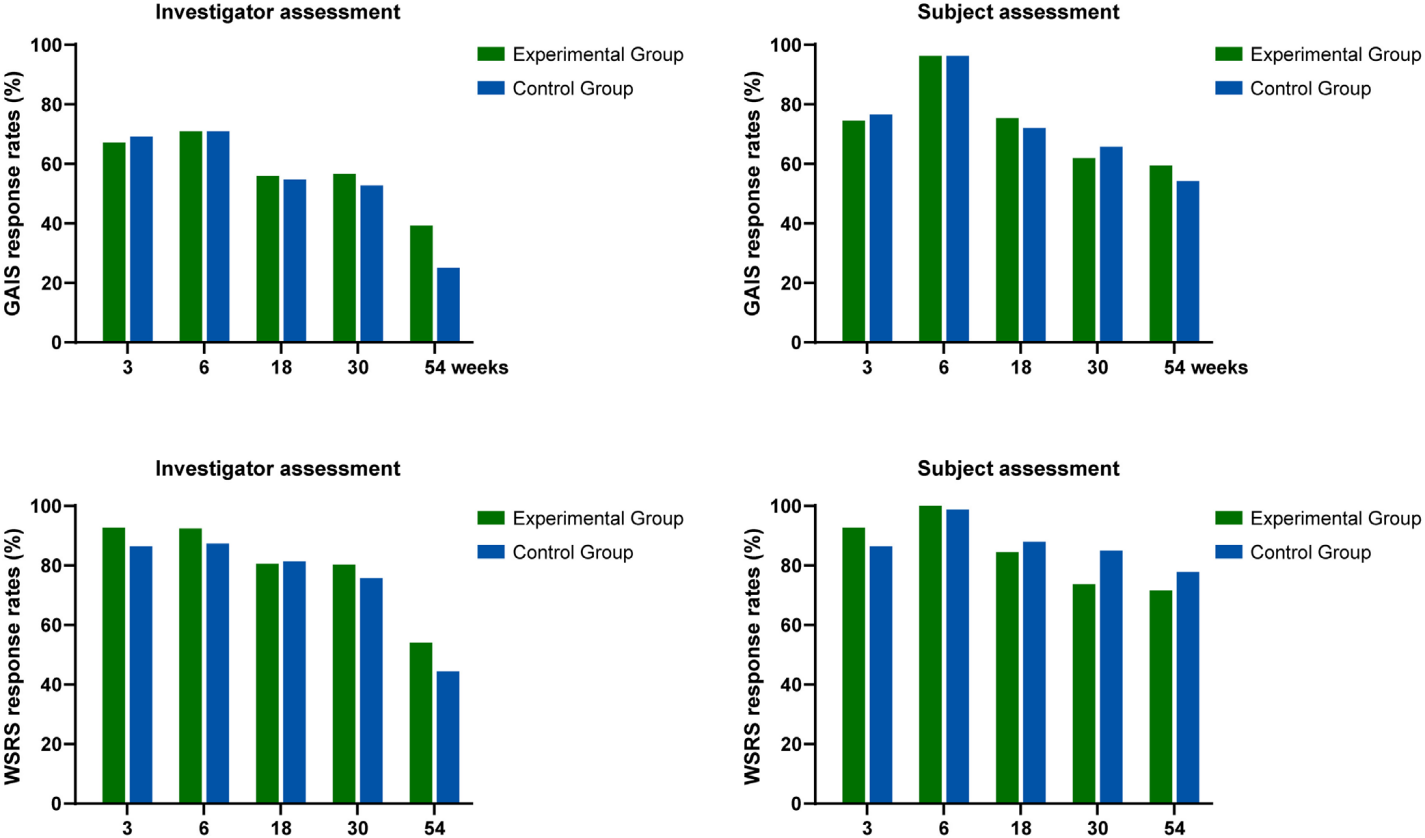
GAIS/WSRS response rates at Week 3, 6, 18, 30, and 54 assessed by independent evaluators and subjects in the FAS population.

### 3.4 Safety

Based on the SS analysis, a total of 403 AEs were reported among 61 subjects in the experimental group, corresponding to an incidence rate of 69.32%. In the control group, 349 AEs were reported among 50 subjects, with an incidence rate of 56.82%. AEs considered related to the investigational device primarily consisted of local injection‐site reactions, including swelling, edema, and erythema. Such events were reported in 26 subjects (29.55%) in the experimental group and 20 subjects (22.73%) in the control group, with no statistically significant difference between groups (*p* > 0.05). Two serious adverse events occurred during the study, namely multiple colorectal polyps and cataract, both of which were assessed as unrelated to the investigational device. Throughout the trial, no clinically meaningful abnormalities were observed in laboratory test results or in vital signs, including blood pressure, pulse rate, or body temperature.

## 4 Discussion

PLLA is a synthetic, biodegradable polymer approved by the United States Food and Drug Administration (FDA) [19]. Owing to its excellent biocompatibility, bioresorbability, and favorable mechanical properties, PLLA was initially developed as a resorbable suture material and has since been extensively utilized in aesthetic medicine and soft‐tissue regeneration [20, 21]. Unlike conventional fillers that primarily achieve immediate correction through physical volumization, PLLA functions as a biostimulatory agent [22]. Following injection, PLLA microspheres elicit a controlled foreign‐body response that activates macrophages and fibroblasts, thereby promoting sustained neocollagenesis, particularly Type I and Type III collagen deposition, along with extracellular matrix remodeling [23, 24]. This regenerative process results in gradual and physiologic restoration of soft‐tissue volume. Clinical improvements typically become evident within 4–8 weeks, peak at approximately 3–6 months, and may persist for up to 12 months [25, 26]. Several clinical studies have demonstrated the efficacy and safety of PLLA fillers for the correction of NLFs. Palm et al. conducted PLLA injections in 80 patients with nasolabial fold wrinkles and followed them for 48 weeks, reporting marked improvements in nasolabial fold appearance and wrinkle severity scores [27]. Brandt et al. compared the efficacy of PLLA with collagen injections for the correction of nasolabial fold wrinkles and demonstrated that PLLA achieved significantly greater clinical improvement than collagen, with treatment effects sustained for up to 25 months [28]. However, treatment outcomes may vary depending on formulation‐specific characteristics, highlighting the need for continued optimization and rigorous clinical evaluation of novel PLLA products.

In this double‐blind, randomized, non‐inferiority controlled clinical trial, we systematically compared the efficacy and safety of the novel PLLA filler HYZ with the marketed product Löviselle for the correction of NLFs. The results showed no statistically significant differences between groups in GAIS response rates. With respect to WSRS outcomes, the experimental group demonstrated an overall trend toward higher response rates than the control group. Notably, independent third‐party assessments at Weeks 30 and 54 revealed significantly greater response rates in the experimental group, suggesting that HYZ may also provide favorable clinical performance during the mid‐ to long‐term collagen remodeling phase. Collectively, these findings indicate that HYZ effectively improves NLF severity with sustained efficacy through 54 weeks and is non‐inferior to Löviselle according to the prespecified non‐inferiority margin. In terms of safety, the overall incidence of adverse events was comparable between groups, with similar safety profiles and no significant between‐group differences. Most events were mild to moderate, injection‐related local reactions, such as swelling, edema, and erythema, indicating that HYZ exhibits acceptable tolerability comparable to the reference product [29].

From a materials science perspective, the PLLA microspheres used in HYZ have been optimized with respect to several critical physicochemical properties. Compared with Löviselle (particle size range, 20–50 μm), HYZ employs microspheres with a tightly controlled size distribution of 25–32 μm (D50 ≈ 25 μm) and a distribution span of less than 1.0, indicating a narrower particle size range and greater dispersity uniformity. This characteristic may increase the surface area of contact between the microspheres and the surrounding tissue, potentially reducing localized post‐injection aggregation and promoting a more homogeneous distribution within the tissue. This may contribute to favorable aesthetic outcomes, although this proposed mechanism remains speculative and requires further investigation [30]. In addition, HYZ offers practical advantages in terms of clinical handling. Löviselle typically requires at least 30 min of pre‐injection reconstitution, whereas HYZ can be reconstituted immediately before administration, achieving complete suspension within approximately 1 min. A previous study has demonstrated that PLLA microspheres (Sifuyan, particle size range: 40–50 μm), which are manufactured by the same company as HYZ using the same manufacturing process and technology, can rapidly hydrate to form a homogeneous suspension [31]. The reconstituted suspension demonstrates favorable stability, with a low tendency for microsphere aggregation or sedimentation, which may facilitate clinical handling and contribute to consistent dosing during injection [32]. In addition, the HYZ series is available in three PLLA dose formulations (45, 75, and 150 mg), providing flexibility in clinical application and allowing individualized treatment planning based on NLFs severity and patient‐specific facial anatomy.

This study has several notable strengths: (1) The large sample size and the double‐blind, randomized, non‐inferiority design minimized selection and assessment bias to the greatest extent possible. (2) The 54‐week follow‐up period comprehensively covered the critical time window for PLLA‐induced collagen regeneration, thereby providing high‐quality evidence regarding the durability of efficacy and long‐term safety. Nevertheless, the limitation of this study should be discussed: The study population consisted predominantly of female participants, which may limit the generalizability of the findings to broader populations. Future studies including more diverse cohorts and extended follow‐up durations are warranted to further strengthen the clinical evidence supporting the efficacy and safety of this novel PLLA filler.

## 5 Conclusion

In this 54‐week randomized, double‐blind, non‐inferiority controlled trial, the novel PLLA filler (HYZ) demonstrated non‐inferior efficacy to Löviselle for NLF correction. No statistically significant difference was observed in the incidence of adverse events between the two groups, supporting the acceptable safety of HYZ. Its optimized microsphere uniformity, immediate reconstitution capability, and multi‐specification design not only enhance the convenience of clinical application but also provide a new direction for the continued refinement of PLLA materials.

## Author Contributions

Conceptualization: F.C. and Q.M. Methodology: L.Z., A.C., S.H., Z.L., and J.S. Validation: Y.D., H.G., Y.Y., X.W., L.C., and C.L. Formal analysis: L.W., J.G., X.Z., W.C., P.Y., and J.L. Investigation: J.S., Y.S., X.Q., Q.H., X.G., and Y.q.Y. Data curation: Z.L., L.Z., S.H., and A.C. Writing – original draft preparation: Z.L. and Q.M. Writing – review and editing: Z.L., F.C., J.S., A.C., and Q.M. Visualization: Z.L. and S.H. Supervision: J.S., L.Z., F.C., and Q.M.

## Funding

The Jilin Scientific and Technological Development Program, Grant number: 20250601014RC. The Jilin Provincial Special Program for Health Research Talents, Grant number: 2024SCZ55.

## Ethics Statement

The clinical trial protocol was approved by the China‐Japan Union Hospital of Jilin University Ethics Committee (No. 2021122909). The protocol was approved by the Yanbian University Hospital Ethics Committee (No. 2022‐004‐01). The protocol was approved by the Shulan (Hangzhou) Hospital Ethics Committee (No. 2022002). The protocol was approved by the Tonghua Central Hospital Ethics Committee (No. QX‐2022‐002).

## Consent

For all personal information used in this article, for example, personal images, a consent for publication has been obtained.

## Conflicts of Interest

The authors declare no conflicts of interest.

## Data Availability

The data that support the findings of this study are available from the corresponding author upon reasonable request.
